# Dimer Interface Organization is a Main Determinant of Intermonomeric Interactions and Correlates with Evolutionary Relationships of Retroviral and Retroviral-Like Ddi1 and Ddi2 Proteases

**DOI:** 10.3390/ijms21041352

**Published:** 2020-02-17

**Authors:** János András Mótyán, Márió Miczi, József Tőzsér

**Affiliations:** 1 Department of Biochemistry and Molecular Biology, Faculty of Medicine, University of Debrecen, 4032 Debrecen, Hungary; miczimario@med.unideb.hu; 2 Doctoral School of Molecular Cell and Immune Biology, University of Debrecen, 4032 Debrecen, Hungary

**Keywords:** retrovirus, retrovirus-like, protease, retroviral protease, dimerization, comparative analysis, contact map, DNA damage-inducible protein, Ddi1, Ddi2

## Abstract

The life cycles of retroviruses rely on the limited proteolysis catalyzed by the viral protease. Numerous eukaryotic organisms also express endogenously such proteases, which originate from retrotransposons or retroviruses, including DNA damage-inducible 1 and 2 (Ddi1 and Ddi2, respectively) proteins. In this study, we performed a comparative analysis based on the structural data currently available in Protein Data Bank (PDB) and Structural summaries of PDB entries (PDBsum) databases, with a special emphasis on the regions involved in dimerization of retroviral and retroviral-like Ddi proteases. In addition to Ddi1 and Ddi2, at least one member of all seven genera of the *Retroviridae* family was included in this comparison. We found that the studied retroviral and non-viral proteases show differences in the mode of dimerization and density of intermonomeric contacts, and distribution of the structural characteristics is in agreement with their evolutionary relationships. Multiple sequence and structure alignments revealed that the interactions between the subunits depend mainly on the overall organization of the dimer interface. We think that better understanding of the general and specific features of proteases may support the characterization of retroviral-like proteases.

## 1. Introduction

Retroviral aspartic proteases, also referred to as retropepsins, belong to family A2 of aspartic proteases. The most characteristic member of this family is the protease (PR) of human immunodeficiency virus type 1 (HIV-1). The viral protease plays a significant role in the replication cycles of retroviruses by processing the viral Gag and Gag-Pol polyproteins. This limited proteolysis is an essential event of the viral life cycle; thus, the viral proteases have become important targets of antiviral therapies.

**Figure 1:**
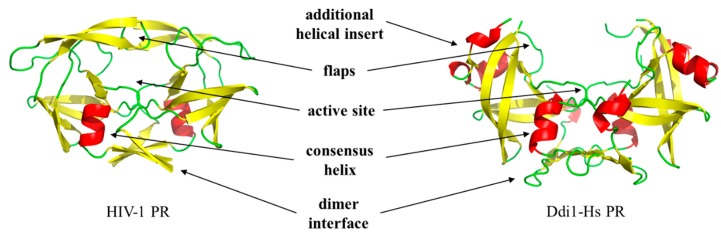
Retroviral and DNA damage-inducible (Ddi) proteins share their main characteristics. Overall structures of human immunodeficiency virus type 1 (HIV-1, PDBID: 5HVP) and human DNA damage-inducible protein 1 ((Ddi1-Hs) (PDBID: 3S8I) proteases are shown. Arrows show important regions, with the exception of the additional α-helical insert, these regions are known to contribute to dimerization.

Retropepsins share their main characteristics. First, they use the same catalytic mechanism, and hydrolysis of the peptide bond is catalyzed by a dyad of highly conserved catalytic Asp residues located in the consensus D-T/S-G-A active site motif. The structural similarities of retropepsins to aspartic protease pepsin implied that they are evolutionarily related to each other. However, in contrast to the bilobal monomeric pepsin, retroviral proteases function as homodimers, and the dimerization is a prerequisite for their activity [1,2]. In addition to the active site motif, other structurally important regions, which are involved in dimer formation, are also shared by the PRs as follows: (i) flap region, (ii) active site loop residues (‘fireman’s grip’ interactions), (iii) dimer interface platform at the bottom of the homodimeric protease, and (iv) consensus α-helix near the C-terminus of the enzyme (Figure 1). 

Numerous eukaryotic organisms have been found to express homologs of retrovirus and retroelement PRs, which evolved from their ancestors and which were retained during their evolution [3,4]. Despite the low sequence identity, retropepsins exhibit high structural similarity, suggesting that the eukaryotic homologs may also share their overall fold and main features with retropepsins. Substantial structural information is available for DNA damage-inducible protein 1 and 2 (Ddi1 and Ddi2, respectively), which are eukaryotic proteins containing a retroviral-like aspartic protease domain [5,6,7,8].

Since the report of the first retroviral PR structures in 1989 [9], several protease structures have been solved, and with the exception of the *Epsilonretrovirus* genus, the structure of at least one member of other six retrovirus genera has already been determined (Table 1A). In the Protein Data Bank (PDB) database, lentivirus PRs are overrepresented, and HIV-1 PR is the most extensively studied member of the *Retroviridae* family [10] (Table 1A). Although numerous retroviral PRs have been characterized in vitro to date, no experimental data are available for some PRs regarding their structure or activity, e.g., bovine immunodeficiency virus (BIV), caprine arthritis encephalitis virus (CAEV), Maedi visna virus, jaagsiekte sheep retrovirus (JSRV), or squirrel monkey retrovirus (SMRV) [11].

Compared with the available data for retroviral PRs, the structures of only few retroviral-like aspartic PRs have been experimentally solved to date. The protease domain of *Saccharomyces cerevisiae* Ddi1 protein (Ddi1-Sc) was the first retroviral-like PR for which its structure was determined by X-ray crystallography [5], and the crystal structures of human Ddi1 (Ddi1-Hs), *Leishmania major* Ddi1 (Ddi1-Lm) and human Ddi2 proteases (Ddi2-Hs) were later reported (Table 1B).

Despite the low sequence identity between the target and template structures, retroviral and retroviral-like PRs exhibit high structural similarity [5], which makes the homology modeling of retroviral-like PRs possible. Before the deposition of the first Ddi1-Lm crystal structure to the PDB in 2017 [8], model structures were previously prepared for Ddi1-like PR of *L. major* [5,12,13], and the PRs of *Schistosoma mansoni* and *Hymenolepis microstoma* [13].

It is known that dimerization is an obligate requirement for retropepsin activity. Using substrate-dependent methods, dimer stabilities can be investigated in vitro by determining the urea concentration leading to a 50% loss in enzymatic activity (UC_50_), or the apparent dimer dissociation constant (K_dapp_) can be determined by measuring enzyme activity at increasing enzyme concentrations, the consequence of less-efficient dimerization is a decreased activity at lower enzyme concentrations. Using kinetic assays, dimer stabilities have already been determined only for some retroviral PRs (Table S1), including wild-type and mutant HIV-1 PRs [44,50,51,52,53], HIV-2 PR [54], xenotropic murine leukemia virus-related virus PR (XMRV PR) [44], human foamy virus PR (HFV PR) [55], human T-lymphotropic virus type 1 PR (HTVL-1 PR) [56], avian myeloblastosis virus (AMV) and Mason–Pfizer monkey virus (MPMV) PRs [53]. Substrate-independent methods are also available for the investigation of dimerization, including thermal denaturation, analytical ultracentrifugation, or circular dichroism [53,57,58,59].

Functional studies have already revealed the importance of Ddi-like proteases. Studies on wild-type and active site mutant Ddi1-Lm proteins revealed changes of the secretion phenotype, and their sensitivity to HIV PR inhibitors also implied the existence of catalytic activity [60]. Studies on Ddi1-Sc PR provided evidence for its proteolytic activity, which was found to be required for sufficient checkpoint regulation [61] and to contribute to protein secretion [62], DNA replication stress response [63], and DNA-protein crosslink repair [64]. In *Caenorhabditis elegans*, Ddi1 expression was found to be induced by proteasome dysfunction; furthermore, results proved that the catalytic activity of Ddi1 PR is necessary for protein activation [65], and involvement of PR activity in Nrf1 processing was also demonstrated for human Ddi2 PR [66,67]. Protease domain of Ddi1-Hs protein was found to undergo post-translational modification, ubiquitination sites were identified in the proximities of the active site (K192) and the dimer interface (K382) [68], but effect of these modifications of protease function has not been elucidated. Among the Ddi-like PRs, proteolytic activity of a recombinant protein was proved in vitro only for Ddi1-Lm [12]. Despite using various methods (e.g., screening an HEK293 cell line-derived peptide library or cleavage reactions by oligopeptide or protein substrates), neither the autoproteolytic activity (cis-activity) nor cleavage of any target protein (trans-activity) has been observed for purified Ddi2-Hs [6] and Ddi1-Sc [5,7] proteases. These studies implied that putative specific factors or determinants may be necessary for PR activation, which remained to be elucidated. 

We propose that in addition to putative specific cellular factors, special structural features may also be important determinants of dimerization. Because of missing experimental data, we investigated dimerization purely based on the structural information currently available in the PDB and PDBsum databases and compared the main characteristics of retroviral PRs and the eukaryotic homolog Ddi1/Ddi2 PRs. To our knowledge, comparative analyses have been conducted only for retroviral PRs [9,69,70,71] and Ddi1-like PRs [13], but retroviral and non-viral PRs have not been compared in such analyses. While comparative analyses of retroviral PRs may support understanding of mutational capacity of HIV-1 PR and resistance development [70], exploring general and specific features of retroviral and retroviral-like PRs may support characterization of retroviral-like PRs with unknown structures and the identification of efficient inhibitors e.g., against Ddi2-Hs PR [67]. Here we describe a comparison of overall structural structures and contact maps, with a special emphasis on the regions involved in dimerization and the correlation of the key features with the evolutionary relationships.

## 2. Results

### 2.1. Phylogenetic Analysis

Evolutionary relationships were investigated by multiple alignment of PR sequences and structures. In the comparison, all seven genera of exogenous retroviruses were represented, and a previously prepared set of retroviral PRs [70,72,73] was complemented with eukaryotic Ddi1 and Ddi2 retroviral-like PRs with experimentally determined structures. The result of the sequence-based analysis is shown later in the Discussion.

**Figure 2:**
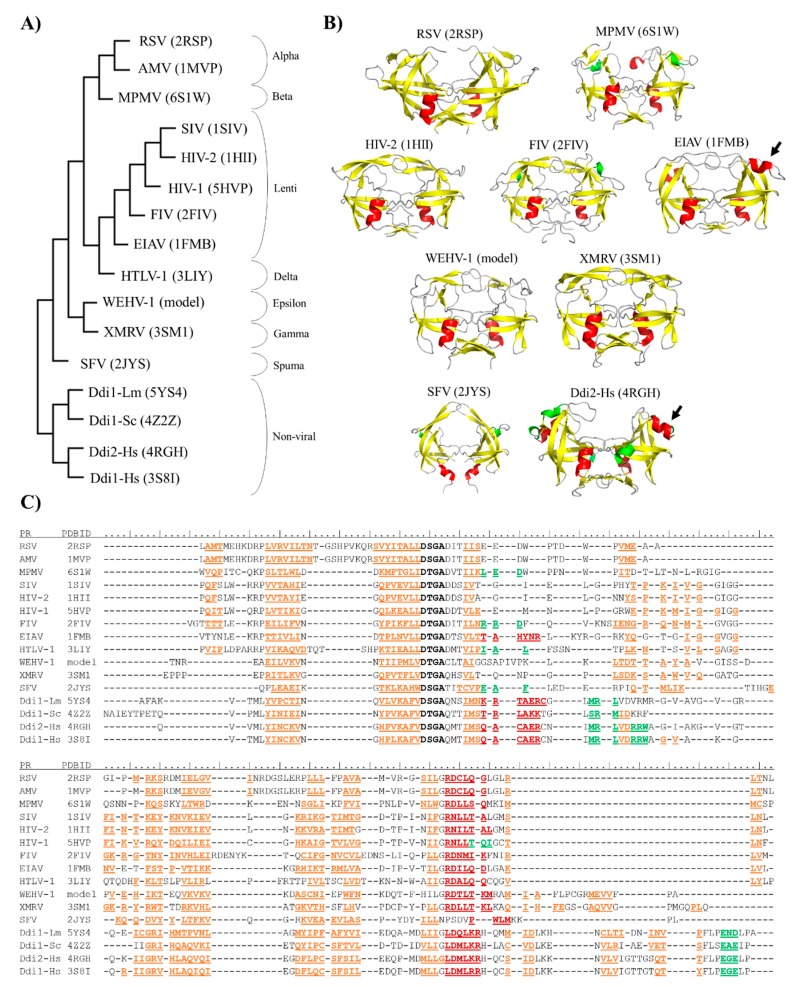
Structure-based phylogenetic analysis of proteases. (**A**) Phylogenetic tree was prepared via the multiple alignment of PR structures (PDBIDs are shown) using the mTM-Align web server. (**B**) Overall structures of representative PRs are shown, PDBIDs are also indicated. Homology model is shown for Walleye epidermal hyperplasia virus type 1 (WEHV-1) PR, homodimeric equine infectious anemia virus (EIAV) and simian foamy virus (SFV) PRs are shown by aligning the monomers to a homodimeric HIV-2 PR structure (PDBID: 1HII). Other structures are represented based on their crystal structures. Structures are colored according to their secondary structures: β-strand, yellow; α-helix, red; 3_10_-helix, green. Arrows indicate additional α-helical inserts. (**C**) Multiple structure alignment of proteases is shown according to the results of analysis performed using mTM-Align web server. Sequences of aligned structures are colored according to the arrangement of secondary structural elements: β-strands, orange; α-helix, red; and 3_10_-helix, green. Active site motif residues are shown in bold. Secondary structural elements are shown according to the Dictionary of protein secondary structure (DSSP) images of PDB, and based on homology model structure for WEHV-1 PR.

A structure-based comparison was also performed, at least one member of each retrovirus genus was represented (Figure 2A). Coordinate files of most retroviral PRs and Ddi eukaryotic homologs were downloaded from the PDB database, with the exception of epsilonretrovirus PRs (Table 1), whose structures has not been determined experimentally to date. Therefore, a coordinate file for a homodimeric Walleye epidermal hyperplasia virus type 1 (WEHV-1) PR was obtained by automated homology modeling (Figure 2B), using the SWISS-MODEL web server, and the proposed structure was used for multiple structure alignment. The structure-based phylogenetic tree was generated using the mTM-align server based on pairwise template modeling scores [74].

The phylogenetic relationships revealed by the multiple structure alignment closely resembled those obtained by the sequence-alignment, and they were in good agreement with those determined using whole viral sequences [75]. Gamma- and epsilonretroviruses were found to be distantly related to lentiviruses, alpha-, beta- and deltaretroviruses, whereas spumaretroviruses exhibited closer relationships with the non-viral Ddi proteins (Figure 2A). The structural characteristics of proteases showed no full correlation with the complexities of retroviral genomes because alpha-, beta-, gamma- and epsilonretroviruses have simple genomes, whereas lentiviruses, delta- and spumaretroviruses have complex genomes.

Based on the phylogenetic trees, we assumed that the structural characteristics correlate with the evolutionary relationships of the proteases. Thus, the results of structure-based alignment (Figure 2C) were used to explore general similarities and differences in detail by focusing mainly on the regions that are important determinants of dimerization.

### 2.2. Dimer Interface Organization

First, we compared the overall organization of the dimer interface, which is a β-sheet platform at the bottom of most PRs (Figure 1). Based on the known structures, three main types of dimer interfaces can be differentiated, and we used the following classification of the dimer interfaces.

**Figure 3:**
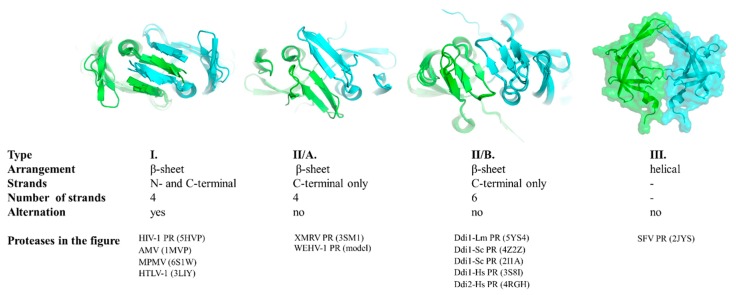
Classification of dimer interfaces. Dimer interfaces are represented according to selected structures, PDBIDs are shown for each protease. Homology model is shown for WEHV-1 PR. Structure of homodimeric SFV PR was proposed by aligning the monomers to a homodimeric HIV-2 PR structure (PDBID: 1HII). Subunits are colored by green and cyan.

The type I dimer interface consists of alternating N- and C-terminal strands and corresponds to the prototypic interface of lentivirus (e.g., HIV-1), betaretrovirus (e.g., MPMV-1), alpharetrovirus (e.g., AMV-1) and deltaretrovirus (e.g., HTLV-1) proteases (Figure 3).

The type II interface contains exclusively C-terminal β-strands that are not interdigitated. Based on the number of strands, this interface type can be subdivided into two subgroups. Four- and six-stranded dimer interfaces can be differentiated, as described for XMRV and Ddi1 PRs, respectively (Figure 3). 

Similarly to type II, the type III interface also exclusively consists of C-terminal regions but exhibits remarkably different organization. Specifically, the regions forming the interface have a helical arrangement instead (Figure 3). To date, the simian foamy virus (SFV) is the only retrovirus that was experimentally proved to have a PR displaying this unusual mode of dimerization [76].

The orientation of the strands in the β-sheet platforms was previously found to depend on the overall organization of the interface [5,46], and both the four- and six-stranded dimer interfaces showed rotations of the strands around the dimer axis compared to those of HIV-1 PR (Figure 3). The rotation of the strands relative to the enzyme’s core is different in Ddi1 compared with that in HIV-1 PR, but it is similar to the topology of the β-sheets (approximately 45° rotation) of non-viral aspartic protease pepsin [2].

The gammaretrovirus XMRV PR is the only retroviral PR that was found to contain a four-stranded dimer interface [46], whereas crystal or solution structures have not revealed such an interface in other retroviral PRs to date. We have prepared a homology model for the WEHV-1 PR, and the results of both the secondary structure prediction and template search suggested highest similarity to XMRV PR, and the same dimer interface organization. Thus, we propose that WEHV-1 PR (and WDSV PR, as well) shares the same four-stranded dimer interface organization as XMRV PR (Figure 3).

Of the studied proteases, only Ddi PRs were found to have six-stranded dimer interfaces (Figure 3), and based on the currently available structural data this dimer interface organization is not characteristic of retroviral PRs. Analysis of additional enzymes may reveal whether this dimer interface organization is a specific feature of eukaryotic retroviral-like PRs.

Structural and biochemical studies also suggested that SFV PR has a specific mode of dimerization that is significantly different from that of other retroviral and retroviral-like PRs [76]. Similarly to epsilonretrovirus, gammaretrovirus and Ddi1/Ddi2 PRs, the N-terminal region of SFV PR is not involved in dimer formation because the presence of N- and C-terminal proline residues makes the formation of dimer interface strands unfavorable [37,76]. In the solution structure of SFV PR, a short α-helix is present instead of a C-terminal β-strand [37], and it does not overlap with the consensus helix of HIV-1 PR. The helix in SFV PR near the C-terminus is in the same position at which a β-strand of the dimer interface is found in the HIV-1 PR structure (Figure 2C). In agreement with this, our secondary structure predictions also implied that foamy virus PRs may have unusual dimer interface (Figure S1). None of the applied algorithms predicted the presence of N- or C-terminal terminal β-strands that correspond to the strands of type I or type II dimer interfaces. Interestingly, the helical arrangements of the C-terminal regions were predicted, but this extension is not visible in the solution structure of SFV PR (Figure 3).

A putative homodimer is represented for SFV PR in Figure 2B. Similarly to a previously proposed dimer [76], this model should only be considered hypothetical because the full-length interface is not visible in the solution structure, and the conformations of the longer helical extensions are hardly predictable without proper template structures. Interestingly, the involvement of C-terminal regions in dimer formation also appears to be unique. In vitro activity assays revealed that both the full-length (1–143) and C-terminally truncated (1–101) forms of the protease possess enzyme activity [37,76]. Based on this finding, the elongated C-terminal regions contribute to dimer stabilization. However, they can provide lower dimer stability as compared to HIV-1 PR (Table S1), are not prerequisites for enzyme activity.

Altogether, these results prove the existence of a type III dimer interface that provides dimerization without N- or C-terminal β-strands. This type of interface platform is significantly different from that of retroviral and Ddi1/Ddi2 PRs, and to date appears to be a unique feature of foamy virus PRs.

### 2.3. Intermonomeric Interactions

**Figure 4:**
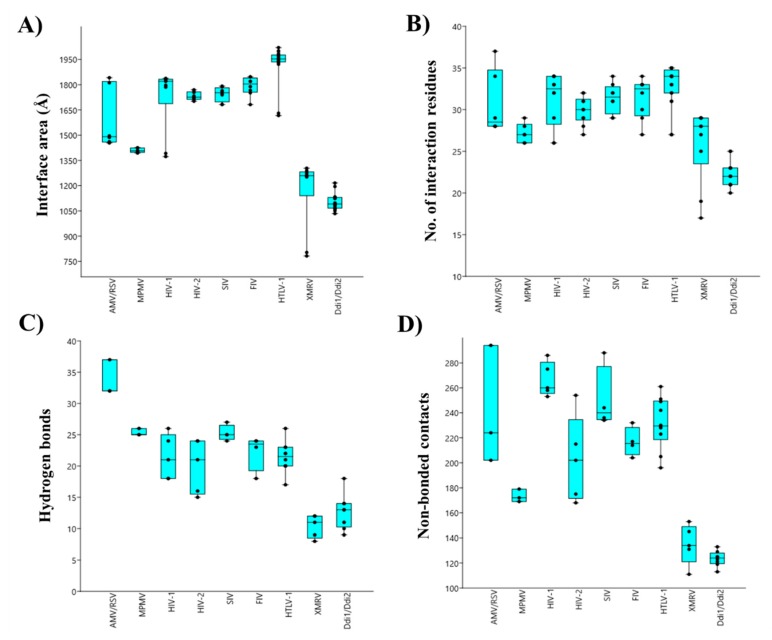
Comparison of overall contact maps. Boxplots represent the means of the total interface area (**A**), number of interface residues (**B**), number of H-bonds (**C**) and number of non-bonded contacts (**D**). Data were plotted by using PAST v3.26 software. Error bars represent SD. Statistical analysis was performed using ANOVA (Mann–Whitney pairwise) algorithm, raw p values are shown in Table S3.

Contact maps of homodimeric PRs were obtained from the PDBsum database, and the areas of intersubunit interfaces (Å) and the total numbers of interface residues, H-bonds and non-bonded contacts were compared (Figure 4). The investigated parameters were found to vary among the different PRs, exhibiting correlations with the phylogenetic relationships. It is important to note that the numbers of currently available structural data are different, and for some proteases, only few structures are available (e.g., AMV, Rous sarcoma virus (RSV), and equine infectious anemia virus (EIAV) PRs); therefore, differences of sample numbers were considered while choosing algorithm for statistical analysis. The coordinate files used for the analysis are listed in Table S2.

The contact densities of XMRV and Ddi PRs were found to be similar but lower than those of the other studied PRs. The difference was statistically significant in almost all cases (Table S3). Although, a larger interface area and higher interface residue number were determined for XMRV PR, the numbers of H-bonds and non-bonded contacts were more comparable with those of Ddi proteins. The contribution of the β-sheet platform to dimer formation is not exclusive, but it provides the main part of the intersubunit interactions. The observed differences of contact densities exhibited obvious correlations with the organization of the dimer interface. Specifically, the contact numbers were significantly lower in XMRV and Ddi PRs possessing four- and six-stranded type II interfaces, respectively, than in other studied PRs containing type I interface (Figure 4). Data for homodimeric foamy virus PRs are not available currently in PDBsum database, thus, spumaretrovirus PRs were not included in the comparison.

The values determined for HIV-1, HIV-2, SIV, and FIV PRs were highly similar, indicating that lentivirus PRs share comparable intermonomeric contacts (Figure 4). Within the *Lentivirus* genus we observed statistically significant differences only in some cases (mainly for the number of non-bonded contacts), but the overall contact densities resembled each other (Table S3).

Values determined for HTLV-1 PR were also similar to those of lentivirus PRs, but a larger interface area and smaller density of non-bonded interactions were observed compared to HIV-1 PR (Figure 4), while the numbers of interacting residues and H-bonds were not significantly different (Table S3). We observed that XMRV PR has significantly lower contact density than HTLV-1 PR, which implied higher in vitro dimer stability for HTLV-1 PR. Despite this, the relatively higher K_dapp_ indicated that HTLV-1 PR has lower dimer stability than XMRV PR (Table S1). Therefore, the overall contact densities may not be obviously in agreement with the in vitro dimer dissociation constants, but the available information is limited.

Interestingly, alpharetrovirus PRs were found to have significantly higher number of intermonomeric H-bonds than other proteases, whereas other studied parameters were more comparable (Figure 4). The structure alignment illustrated that AMV and RSV PRs are longer than HIV-1 PR because of a two-residue-long C-terminal extension (N123 and L124 in each structure). Additionally, they contain a short insertion between the β-strands corresponding the sixth and seventh strands of HIV-1 PR, and consequently, the loop connecting the β-strands is closer to the dimer interface, permitting intersubunit interactions at this site. These interactions are missing from the bottom of HIV-1 PR (Figure S2) and are responsible in part for the higher number of H-bonds in AMV and RSV PRs.

In addition to the overall differences and similarities of intermonomeric contacts, we analyzed the regions that are known to contribute to dimer formation in detail, and investigated differences in (i) ‘fireman’s grip’, (ii) the salt bridges formed near that catalytic site, (iii) the consensus α-helix and iv) the flaps.

### 2.4. ‘Fireman’s Grip’

It is known that the Thr/Ser residues present in the conserved D-T/S-G-A active site motif of retroviral PRs are essential for dimer formation and stabilization via intermonomeric interactions referred to as ‘fireman’s grip’ [57]. The ‘fireman’s grip’ interactions provided by the hydroxyl group of Thr can be substituted by that of Ser, but higher K_dapp_ values were determined for HIV-1 and AMV PRs containing Ser in this position, proving the contribution of the Thr side chain to dimer formation and stabilization [53,57,77]. 

We compared the active site motif sequences to investigate the conservation of Thr or Ser residues in retroviral PRs and in non-viral Ddi proteins. 

**Figure 5:**
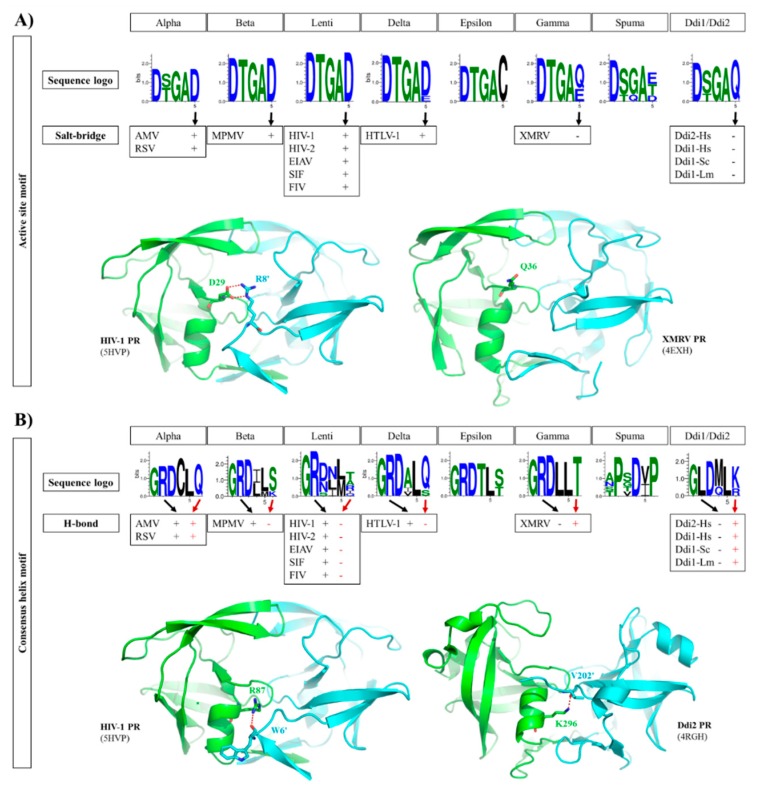
Intermonomeric interactions at the active site and consensus helix. (**A**) Sequence logos of active site motifs are shown. The formation of a salt bridge between the active site motif (Glu residue) and an N-terminal residue of the other subunit is denoted by ‘+’, whereas the absence of a salt bridge in the corresponding position is denoted by ‘-‘, as represented by structures of HIV-1 and XMRV PRs. (**B**) Sequence logos of consensus α-helices are shown. The involvement of consensus helix residues in H-bond formation is denoted by ‘+’ or ‘-‘. In the consensus helix, positively charged Arg and Lys residues can form H-bonds (red dotted lines) with the N-terminus of the other subunit, as shown for HIV-1 and Ddi2-Hs PRs, respectively. Sequence logos were prepared based on the sequences used for phylogenetic analysis. Residue interactions were determined on the basis of contact maps (PDBsum database) of the structures used for multiple structure alignment (Figure 2A).

We found that Thr is highly conserved in the active site motifs of lentiviruses, beta-, delta-, epsilon- and gammaretrovirus PRs, whereas the residue may be either Thr or Ser in alpharetrovirus, spumaretrovirus, and Ddi PRs (Figure 5A). It was hypothesized that enzymes having looser ‘fireman’s grip’ interactions exhibit lower dimer stability and activity [77]. 

We found that the third residue of the D-T/S-G-A active site motif is highly conserved in both retroviral and retroviral-like PRs (Figure 5A). This glycine is replaced by glutamine in this position exclusively in FFV PR among the studied proteases (Figure S1). The Ddi1-like proteins of platyhelminth parasites were found previously to contain Ser in the active site motif [13], but among the studied Ddi PRs, Thr was found to be present only in Ddi1-Sc PR.

### 2.5. Active Site Motif

In HIV-1 PR and in most retroviral PRs, the consensus D-T/S-G-A active site motif is followed by an Asp residue, which is known to be involved in dimer formation. In HIV-1 PR, a salt bridge is formed between this Asp and an Arg residue at the N-terminus of the other subunit (D29 and R8′ residues, according to the HIV-1 PR numbering) (Figure 5A). Mutagenesis of this Arg residue revealed that substitution by Gln causes only a slight decrease in activity compared with that of wild-type HIV-1 PR, indicating that the ion pair contributes to but is not essentially required for dimer formation [78].

The sequence logos of the active site motif were compared, and we found that the PRs of lentiviruses, alpha-, beta- and deltaretroviruses, which are closely related to each other (Figure 2), exhibit high conservation of the Asp residue forming the salt bridge (Figure 5A), with the exception of BLV PR, which contains Glu in this position. Based on the contact maps, the Asp residue in these enzymes can form a salt bridge with an Arg residue of the other subunit. Interestingly, MPMV PR contains a Lys instead of Arg in its N-terminal region (R8 in HIV-1 PR) (Figure 2), but an ion pair can also be formed between this Lys and the Asp residues in the active site (Figure 5A).

In contrast with this, epsilon-, gamma- and spumaretrovirus, and Ddi PRs contain different residues in this position, namely Cys or Gln in epsilonretrovirus and Ddi2 PRs, respectively. Glu, Gln, and Thr are the most prevalent residues in gamma- and spumaretrovirus PRs, whereas Asp is present only in FFV PR in this position (Figure S1). XMRV and Ddi PRs contain Gln or Glu in this position, and the contact maps revealed no formation of salt bridges in their crystal structures (Figure 5A). In accordance with the literature data [46], mainly hydrophobic contacts are formed between the subunits, which replace the ionic interactions. Based on PDBsum data, H-bonds are not formed by the fifth residue of the active site in these PRs, but non-bonded interactions are formed instead.

Epsilonretrovirus proteases exclusively contain Cys in this position; thus, it can be expected that a salt bridge is not formed by this motif residue. All foamy PRs contain Thr after the consensus active site motif instead of Asp (Figure 5A); thus, based on sequences they also lack the intermonomeric ion pair. These data are in agreement with the higher dimer stability of HIV-1 PR compared with that of XMRV PR (Table S1) because of the contribution of salt bridges to dimer stabilization.

### 2.6. Consensus α-Helix

The structures of homodimeric retroviral and retroviral-like aspartic PRs share the same protease fold. Each subunit consists of mainly β-strands, but the presence of a consensus short α-helix near the C-terminus is also a common feature that encompasses one of the most conserved sequence motifs of these enzymes. This α-helix contains G-R-N motif in HIV-1, HIV-2, and SIV PRs, whereas G-R-D motif is present in other lentivirus PRs and in all other retroviral genera members, excepting spumaretroviruses (Figure 5B). 

In HIV-1 PR, the side chain atoms of R87 residue have H-bond interactions with the main chain atoms of L5′ and W6′ residues [79]. The significance of these interactions was investigated previously via mutagenesis studies. The G86 residue was found to participate in the coordination of active site loop’s conformation, which is necessary for efficient substrate binding and proteolysis. In contrast with this, the neighboring R87, which makes intersubunit contacts, was found to contribute to dimer stabilization, as the R87K mutant protein had impaired ability for dimerization, and it was mainly monomeric in solution [79,80,81]. 

These interactions of the side chain of Arg with the main chain atoms of one or two residues of the other subunit’s N-terminus can be observed in the structures of HIV-1, HIV-2, EIAV, SIV, FIV, AMV, RSV, and HTLV-1 PRs. The side chain of R95 in the G-R-N/D motifs of XMRV PR structures do not interact with the P14′ residues. Structure of WEHV-1 PR was built up based on that of XMRV PR; therefore, the model also revealed no R95 side chain-mediated interaction with the other subunit.

It appears that pattern of interactions between the consensus C-terminal helix and N-terminal residues is different in the case of PRs with type I (e.g., HIV-1 and HTLV-1 PRs) or type II (e.g., Ddi1 PRs) dimer interfaces. Ddi PRs contain a G-L-D-M-L-K/R sequence in the consensus helix, which lacks the Arg residue of the G-R-D conserved motif but contains a downstream positively charged residue (Lys or Arg) that is exposed to the intersubunit surface, such as R87 of HIV-1 PR). This residue can also form H-bonds with the N-terminus of the other subunit (Figure 5B).

The C-terminal α-helix of SFV PR does not overlap with that of HIV-1 PR, as it is located closer to the C-terminus almost where a β-strand is found in the HIV-1 PR structure (Figure 2C). Additionally, the sequence motif of the consensus α-helix is completely different from those of retroviral and Ddi PRs, and no G-R-N/D motif is present in SFV PR. The difference in the sequence, especially the Arg-to-Pro substitution, makes the formation of the helix in the same position unfavorable [37]. This also proves that foamy virus PRs possessing type III interface exhibit significantly different distribution of intermonomeric interactions, which remain to be explored in detail by studies of homodimeric spumaretrovirus PRs.

### 2.7. Flaps

In retroviral PRs, the flaps have mainly β-hairpin conformation. Due to their flexibility they can adopt opened, semi-opened and closed conformations, which conformational changes contribute to substrate binding and product release, as well (Figure S3). Whereas the opened conformation permits ligand binding, in the closed conformation the flaps cover the active site, and in the ligand-bound state they wrap around the substrate (or inhibitor) [82]. 

**Figure 6:**
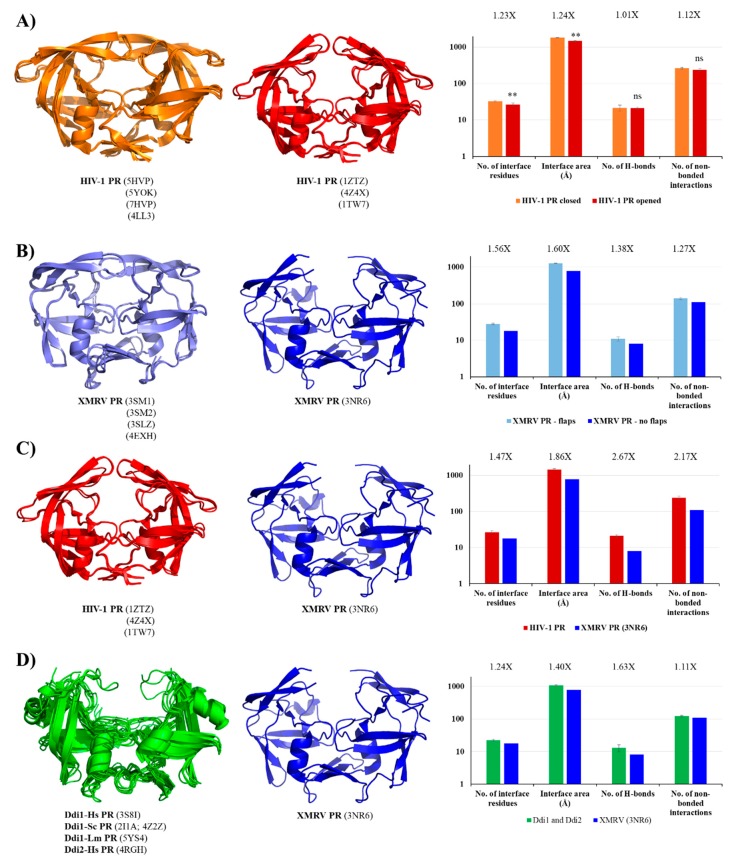
Comparison of contact maps. (**A**) Contact maps of HIV-1 PR structures. Structures and contact numbers are shown for HIV-1 PR in the closed and opened conformations (orange and red, respectively). (**B**) Contact maps of XMRV PR structures. Structures and contact numbers are shown for XMRV PR structures either containing or lacking full-length flaps (light and dark blue, respectively). (**C**) Contact maps of HIV-1 and XMRV PR structures. Structures and contact numbers are shown for the opened conformational HIV-1 PR (orange) and for an XMRV PR lacking full-length flaps in the crystal structure (blue). (**D**) Contact maps of Ddi and XMRV PR structures. Structures and contact numbers are shown for Ddi PRs (green) and for XMRV PR lacking full-length flaps in the crystal structure (blue). In all cases, the structures are represented based on crystal structures. Graphs shows intermonomeric contacts for the homodimers based on the PDBsum database. Fold differences between the values are shown. In figure part (**A**), ** indicates that *p* < 0.01, while ns denotes no statistically significant difference. For figure part (**B**–**D**), where a single XMRV PR structure (3NR6) was used for the comparison, statistical analysis was not performed because the sample number was only 1.

In Ddi PRs, these regions—corresponding to the flaps of HIV-1 PR—adopt less-ordered conformational loops and exhibit higher flexibility; consequently, only one or none of the loops is visible in the known structures [5,6,7,8]. Contact maps proved that both the residue number and area of the interface are higher in the closed conformation. The number of H-bonds was almost identical in the different conformations and the number of non-bonded interactions was slightly lower in the opened conformation in the investigated HIV-1 PR structures (Figure 6A), due to the missing flap-interactions. XMRV PR structures either containing or lacking full-length flaps were also compared, and we found to have remarkably larger interface area and higher contact density if the full-length flaps were present in the structure (Figure 6B), which further proved the role of flaps in the intermonomeric interactions.

In order to investigate whether HIV-1 PR has higher contact density than XMRV PR, we compared the structures of HIV-1 and XMRV PRs in the opened conformation. We found that all studied values were ≥1.5-fold higher for HIV-1 PR, indicating higher contact density (Figure 6C). Although full-length flaps are lacking in the studied XMRV PR structure and they do not contribute to intermonomeric interactions in HIV-1 PR, the observed differences are caused by the characteristics of other regions, mainly by the dimer interface organizations.

In contrast with retroviral PRs, the flaps of Ddi proteins do not contribute to dimerization. These dimers are instead stabilized mainly by intermonomeric interactions in the dimer interface platform [6]. To test this, we compared the contact densities of Ddi proteins with that of XMRV PR lacking full-length flaps. We expected similar contact numbers for the different PRs, because they all have the type II dimer interface and no contacts between the flaps, and the overall interactions were also comparable (Figure 4). Interestingly, we found higher contact densities for Ddi PRs than for XMRV PR (Figure 6D). 

**Figure 7:**
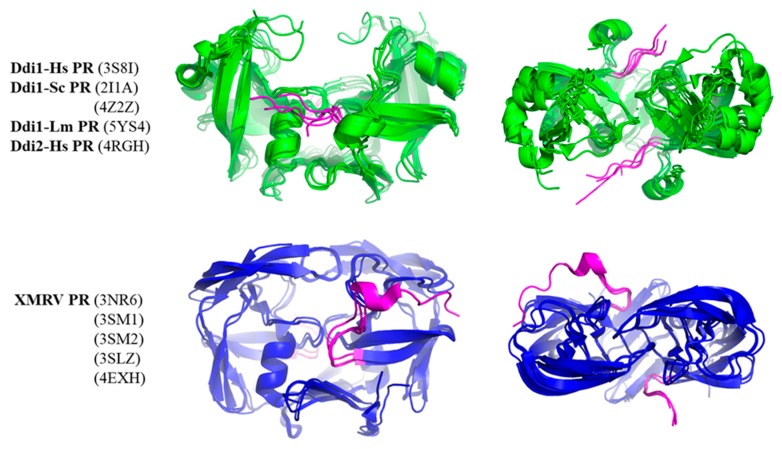
Conformations of N-terminal regions are different in Ddi and XMRV PRs. Structures are shown based on crystal structures. The side and top views are presented. Ddi and XMRV PR structures are indicated in green and blue, respectively, whereas N-termini are colored by magenta in all structures.

Although other regions may also be responsible for the observed differences, we studied whether N-terminal regions could contribute to intermonomeric interactions. Therefore, we compared whether N-terminal regions have different conformations in Ddi and XMRV PRs (Figure 7). In the crystal structures of XMRV PR, the N-terminus is curved in the direction of the flap elbow, but the N-terminal regions in Ddi proteins exhibit extended conformations, being oriented towards the other subunit. Based on the PDBsum data, the N-terminal residues of Ddi proteins also form both H-bonds and non-bonded interactions with the other subunit. In XMRV PR structures, no H-bonds are formed, and the number of non-bonded interactions is lower. Importantly, the involvement of these termini in the dimerization was interpreted in the context of the available crystal structures, but they may have different conformational states in solution based on small-angle X-ray scattering (SAXS) analysis [7].

The interaction of the N-terminus with the other subunit may be relevant in PRs having type II or type III interface because the N-terminal regions are not involved in the formation of the β-sheet platform. Consequently, they may have higher flexibility, and they can possibly form intermonomeric interactions. It is known that N-terminal extensions have unfavorable effects on the activity of HIV-1 PR with a type I dimer interface [83], but the effects of N-terminal regions on the activities of epsilon-, gamma-, and spumaretrovirus and non-viral PRs with type II interface have not been investigated to date. Additionally, autoproteolysis of Ddi proteins has not been reported, and it is possible that the N-terminal regions remain attached, contain no autoproteolytic site, and form a connection between the protease domain and the preceding HDD domain, as observed in the structures of Ddi2-Hs [6] and Ddi1-Sc [7] PRs. Consequently, the possible conformational states of the N-terminal extensions need to be elucidated in future studies to better understand their putative involvement in dimerization.

### 2.8. Additional α-Helical Insert

The presence of an additional α-helical insert was first observed in the crystal structure of EIAV PR [26]. This region, which adopts an α-helical conformation, is located in the flap elbow, near the conserved active site motif (Figure 1). The structural role of this additional helix has been revealed by molecular dynamics simulations suggesting its determinant role in the flexibility of the flaps [8]. Despite the relatively higher rigidity of flap movement, the tips of the flaps have high flexibility in Ddi PRs, and thus, they are not defined fully in most structures by electron density maps.

This additional α-helical region exhibits similarity to the vertebrate counterpart pepsin [26], and it is not characteristic of most retroviral PRs. Therefore, we investigated whether the helicity of this region exhibit characteristic distribution. Although MPMV, HTLV-1, FIV and SFV PRs contain a 3_10_-helix in the corresponding position, this region adopts an α-helical conformation only in EIAV PR among the retroviral PRs, and is present in the structure of Ddi PRs, as well (Figure 2C).

Based on the currently available structural information, the α-helical insert in the flap elbow is present in all known Ddi PR structures. Whereas EIAV PR is the only retroviral protease that contains this additional α-helix, it appears that the presence of this helix is not uniquely characteristic for Ddi PRs.

## 3. Discussion

Structural studies of retroviral PRs have been reviewed previously [9,69,71], and comparative structural analyses of retroviral [70,72,73] and Ddi1-like PRs [13] were also conducted. However, to our knowledge a detailed comparative analysis of retroviral and non-viral PRs has not been reported previously. Therefore, in this work we studied general and specific structural features of retroviral and retroviral-like Ddi1 and Ddi2 PRs, based on structural data currently available in PDB and PDBsum databases with a special emphasis on the regions involved in dimer formation.

In this study, all seven genera of the *Retroviridae* family were represented at least by one member, and eukaryotic Ddi retroviral-like PRs with experimentally determined structures were also included. For retroviral PRs, preparation of a homology model was necessary only in the case of WEHV-1 PR, as the structural coordinates were available for others.

Similarly to the specificity patterns of retroviral PRs [70,72,73], we found that overall structural features also exhibit characteristics distribution in accordance with the evolutionary relationships. The results of multiple sequence and structure alignments were in good agreement with each other and reflected the previously established evolutionary relationships of retroviruses [75]. 

**Figure 8:**
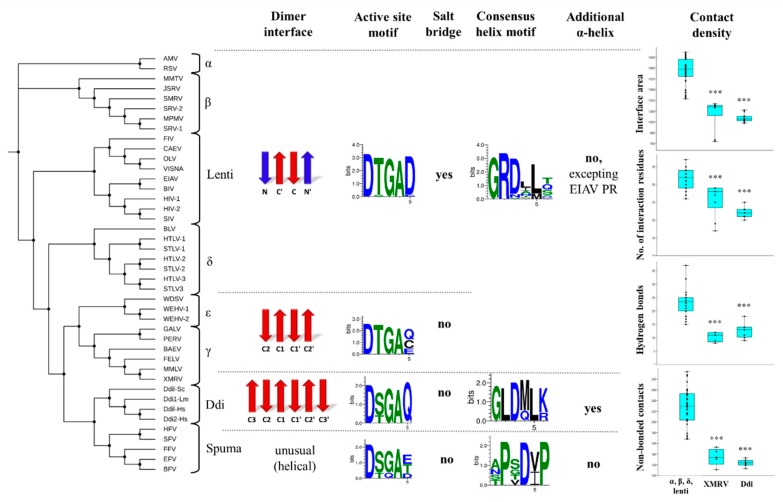
Correlation of structural characteristics with evolutionary relationships. The phylogenetic tree was obtained via multiple alignment of the protease sequences. The names of retroviruses are defined in the list of abbreviations. The features that were characteristic of the different groups are shown horizontally (separated by dashed lines). For dimer interfaces, N- and C-terminal β-strands are indicated in red and blue, respectively. Sequence logos were prepared on the basis of protease sequences of the different groups. The possible occurrence of a salt bridge formed by the fifth residue of active site motif is also shown. The presence of additional α-helical inserts is shown, the 3_10_-helices are not presented in the figure. For comparisons of contact maps, the values are shown based on the PDBsum database for (i) lentivirus, alpha-, beta- and deltaretrovirus PRs, (ii) XMRV PR, and (iii) Ddi proteases. *** denotes statistically significant difference (*p* < 0.001) as compared to the group of lentivirus, alpha-, beta- and deltaretrovirus PRs, determined by using PAST v3.26 software.

We classified the dimer interface platforms into three main groups, based on their overall organization. Types I and II dimer interfaces are built up by N- and/or C-terminal β-strands, whereas type III interface exhibits a completely different structure and helical arrangement of interface-forming termini. We found that the overall organization of the dimer interface varies among retroviral PRs (Figure 8). The type I interface is characteristic of lentiviruses, alpha-, beta- and deltaretrovirus PRs, whereas the type II/A interface is characteristic of epsilon- and gammaretrovirus PRs. Based on the currently available structures, the six-stranded β-sheet platform (type II/B) is characteristic only for non-viral Ddi proteins, and the type III helical interface is a unique feature of spumaretrovirus PRs. We assumed that other eukaryotic retroviral-like aspartic proteases have the type II/B dimer interface, but this must be elucidated via extensive homology modeling of non-viral PRs, which was beyond the scope of this study.

Based on literature data, the dimer interface organization may be one of the key determinants of dimer stability. It was reported previously that the interdigitation of N- and C-terminal strands (as observed in HIV-1 PR with the type I interface) may provide a much higher number of intermonomeric contacts than observed in PRs with the type II dimer interface, in which the C-terminal strands exhibit no alternation (e.g., XMRV PR) [46]. In agreement with this hypothesis, relatively lower UC_50_ and higher K_dapp_ have been reported previously for XMRV PR, implying its lower dimer stability compared with that of HIV-1 PR (Table S1). Our comparison of contact densities revealed a good correlation with this observation because XMRV PR had a significantly smaller interface area and lower number of contacts than other retroviral PRs (excluding spumaretrovirus PRs, which were not included in this comparison). The contact densities of Ddi PRs were highly similar to that of XMRV PR (Figure 8), suggesting relatively lower dimer stability for these non-viral proteins. 

Comparison of active site motifs revealed the conservation of Thr in the ‘fireman’s grip’ of retroviral PRs, excluding foamy virus PRs which contain Ser in this position. Ddi PRs were also found to contain mainly Ser in this position. Given that the fact that the Ser residue in the D-T/S-G-A active site motif can provide looser ‘fireman’s grip’ interactions than Thr [53,57,77], we assumed that Ddi PRs containing D-S-G-A active site motifs may possess lower dimer stability. Nonetheless, other regions also contribute to dimer stabilization; thus, the determinant role of the side chain of Ser or Thr in dimer stabilization is not exclusive.

We found that the PRs with the type I dimer interface, i.e., in lentiviruses, alpha-, beta- and deltaretroviruses, the active site loop contains a highly conserved aspartate residue next to the D-T/S-G-A motif, the residue of which can form a salt bridge with the other subunit. Interestingly, this salt bridge was not observed in PRs possessing type II or III dimer interfaces, whereas in the equivalent positions, they contain no charged residue pairs (Figure 8). The formation of salt bridges implies stronger intermonomeric contacts, but contradictorily, relatively lower dimer stability was measured for HTLV-1 PR (Table S1).

The distribution of the G-R-D consensus helix motif was found to resemble that of the Thr ‘fireman’s grip’ residue. We found that the Arg residue providing an important H-bond with the other subunit [79,80,81] is conserved in the G-R-D consensus helix motifs of all retroviral PRs, excepting spumaretrovirus PRs (Figure 8). This important H-bond is missing in Ddi PRs because they exclusively contain Leu instead of Arg. Interestingly, Ddi PRs contain Arg or Lys residues in their consensus helix, but not in the equivalent position of the helix but close to its C-terminus. These positively charged residues can also form H-bond interactions with the other subunit. The interactions of the consensus helix are also special in foamy virus PRs, because it does not cover the helices of other PRs in its spatial position, and it contains Pro at the position of Arg in the G-R-D motif, denoting an entirely different sequence motif.

The helical arrangement of the region in the proximity of the active site was also studied; however, it does not contribute to dimer formation. We found that the presence of the helix is not a common feature of retroviral PRs. It adopts an α-helical conformation only in EIAV PR, whereas it forms a 3_10_-helix in MPMV, HTLV-1, FIV and SFV PRs. The Ddi PRs of all studied species were found to contain the additional α-helix, but it needs to elucidated in future structural studies whether other eukaryotic retroviral-like PRs share this feature.

Possibly, the presence of the additional α-helical insert may be related to the substantially different substrate binding mode considered characteristic of Ddi PRs. The conformations of flaps are different in Ddi PRs, and they do not resemble those of HIV-1 PR. In the known structures, the flaps make the active site accessible to the solvent. The relatively wider substrate cavity is supposed to be responsible for recognising secondary or tertiary structure—it permits the binding of smaller globular proteins [5,6,8]. Despite the special flap arrangement and larger substrate cavity compared with those in HIV-1 and other retroviral PRs, Ddi1-Lm PR exhibited substrate binding and catalytic activity [12]. A polypeptide substrate has been modelled to the substrate binding cavity of the homodimeric Ddi1-Sc PR, and the structure of the proposed complex indicated that the enzyme can bind a polypeptide, despite the lower number of closer enzyme-substrate contacts [5]. The mode of the putative substrate engagement was revealed by the crystal structure of Ddi1-Sc PR, and because of a crystallization artefact, the N-terminal region of the protein was found to act as pseudo-substrate. Therefore, the flaps did not exhibit a closed conformation, and in this binding mode, the pseudo-substrate was exposed to the solvent [7]. Whereas, the flap elbow of HIV-1 PR provides an opening of the flaps, the additional α-helix affects flap movement in Ddi1-Lm PR [8], and it may be a structural determinant of substrate engagement.

In conclusion, our results suggest that the organization of dimer interface is a main determinant of the intermonomeric interactions, and the distribution of characteristics of the regions involved in dimer formation is in accordance with the evolutionary relationships, not only among retroviral proteases but also relative to retroviral-like Ddi proteases. The homodimerization of Ddi1 and Ddi2 proteases have already been proved either by X-ray crystallography or SAXS [5,6,7,8,84], but characteristics of dimerization, especially in vitro dimer stabilities (e.g., UC_50_ and K_dapp_ values) are available in the literature only for some PRs having type I or type II/A interface, but not for enzymes having type II/B interface organization. Our results imply that non-viral retroviral-like PRs potentially have relatively lower dimer stability compared to retroviral counterparts. We assume that getting better insight into the structural requirements for the dimer formation through the protease domain may help understanding the roles of Ddi-like proteins in proteasomal shuttles and ubiquitination pathways [6,7,63,64,67,68,84,85], even in physiological or pathological conditions. Furthermore, future studies are needed to prove our findings by determining the in vitro dimer stabilities, and correlating the features described in our study with those of additional eukaryotic retroviral-like or endogenous retrovirus PRs.

## 4. Materials and Methods 

### 4.1. Data

Protease sequences were obtained from previous comparative analyses [72,73] and from the UniProt knowledgebase (https://www.uniprot.org) [86]. The identifiers of the studied proteases are listed in Table S4. Coordinate files were downloaded from the Protein Data Bank (http://www.rcsb.org) [87]. Contact maps were downloaded from the PDBsum database (http://www.ebi.ac.uk/pdbsum) [88]. The protease structures used for comparison are listed in Table S2.

### 4.2. Analysis

Multiple sequence alignment was performed using Clustal X v1.83 [89]. Phylogenetic tree was built using the Interactive Tree Of Life web server (iTOL, https://itol.embl.de) [90]. Sequence logos were prepared by WebLogo 3 web server (http://weblogo.threeplusone.com/create.cgi) [91]. Secondary structure predictions were performed using GORIV (https://npsa-prabi.ibcp.fr/cgi-bin/npsa_automat.pl?page=npsa_gor4.html) [92] and JPred4 (http://www.compbio.dundee.ac.uk/jpred) [93] servers. The SWISS-MODEL server (https://swissmodel.expasy.org) was used for homology modeling of WEHV-1 PR [94], using the crystal structure of XMRV PR as template (PDBID: 3SM1). Multiple protein structure alignment was performed by using mTM-align web server (http://yanglab.nankai.edu.cn/mTM-align) [74]. Structural figures were prepared using the PyMOL Molecular Graphics System (Version 1.3, Schrödinger, LLC, Portland, OR, USA). Statistical analysis was performed by the PAST v3.26 software, using Mann–Whitney pairwise algorithm (several-sample tests) [95]. 

## Figures and Tables

**Table 1:** Retroviral and retroviral-like PRs in Protein Data Bank. Coordinate files available in the PDB are shown for retroviral (**A**) and Ddi1/Ddi2 retroviral-like PRs (**B**). Only some representative PDB IDs are presented if >10 coordinate files are available. For HIV-1, only an approximate value is shown, based on a refined search on text-(on ‘HIV-1 protease’) and structure title-search (on ‘HIV-1′ and ‘protease’). Database was accessed in October 2019.

(**A**)					
**Retrovirus Genus**	**Representative Virus**	**Name**	**Number of IDs**	**PDB ID**	**Reference**
Lentiviruses	Human Immunodeficiency virus type 1	HIV-1	>600	5HVP	[14]
				1G6L	[15]
				3PHV	[16]
				1ZTZ	[17]
				4LL3	[18]
				7HVP	[19]
				5YOK	[20]
				4Z4X	[21]
				1TW7	[22]
	Human Immunodeficiency virus type 2	HIV-2	19	1HII	[23]
				5UPJ, 6UPJ	[24]
				2HPE	to be published
				3EBZ	[25]
	Equine infectious anemia virus	EIAV	2	1FMB	[26]
				2FMB	[27]
	Simian Immunodeficiency virus	SIV	7	1SIV	[28]
				1TCW	[29]
				1YTI, 1YTJ, 1YTH, 1YTG	[30]
				1AZ5	[31]
	Feline immunodeficiency virus	FIV	10	4FIV	[27]
				1FIV	[32]
				2FIV, 3FIV	[33]
				5FIV, 6FIV, 1B11	[34]
				2HAH	[35]
				3OGP, 3OGQ	[36]
Spumaretroviruses	Simian Foamy virus	SFV	1	2JYS	[37]
Alpharetroviruses	Avian myeloblastosis virus	AMV	1	1MVP	[38]
	Rous Sarcoma Virus	RSV	2	1BAI	[39]
				2RSP	[40]
Deltaretroviruses	Human T-lymphotropic virus type 1	HTLV-1	10	3LIY, 3LIX, 3LIV, 3LIQ, 3LIN, 3LIT	[41]
				3WSJ, 4YDF, 4YDG	[42]
				2B7F	[43]
Epsilonretroviruses	Walleye epidermal hyperplasia virus type 1	WEHV-1	0	-	-
Gammaretroviruses	Xenotropic murine leukemia virus-related virus	XMRV	5	4EXH	[44]
				3SLZ, 3SM1, 3SM2	[45]
				3NR6	[46]
Betaretroviruses	Mason–Pfizer monkey virus	MPMV	5	6S1U, 6S1W, 6S1V	[47]
				3SQF	[48]
				1NSO	[49]
(**B**)					
**Protein**	**Organism**	**Name**	**IDs**	**PDB ID**	**Reference**
Non-viral (eukaryotic)	*Saccharomyces cerevisiae*	Ddi1-Sc	2	2I1A	[5]
				4Z2Z	[7]
	*Homo sapiens*	Ddi1-Hs	1	3S8I	to be published
		Ddi2-Hs	1	4RGH	[6]
	*Leishmania major*	Ddi1-Lm	2	5YS4, 5YQ8	[8]

## Supplementary Materials

The following are available online at https://www.mdpi.com/1422-0067/21/4/1352/s1.

## Abbreviations

AMV	Avian myeloblastosis virus
BAEV	Baboon endogenous virus
BFV	Bovine foamy virus
BIV	Bovine immunodeficiency virus
BLV	Bovine leukemia virus
CAEV	Caprine arthritis encephalitis virus
Ddi1-Lm	DNA damage-inducible protein 1 (*Leishmania major*)
Ddi1-Sc	DNA damage-inducible protein 1 (*Saccharomyces cerevisiae*)
Ddi1-Hs	DNA damage-inducible protein 1 (*Homo sapiens*)
Ddi2-Hs	DNA damage-inducible protein 2 (*Homo sapiens*)
EFV	Equine foamy virus
EIAV	Equine infectious anemia virus
FELV	Feline leukemia virus
FFV	Feline foamy virus
FIV	Feline immunodeficiency virus
GALV	Gibbon ape leukemia virus
HFV	Human foamy virus
HIV-1	Human immunodeficiency virus type 1
HIV-2	Human immunodeficiency virus type 2
HTLV-1	Human T-lymphotropic virus type 1
HTLV-2	Human T-lymphotropic virus type 2
HTLV-3	Human T-lymphotropic virus type 3
JSRV	Jaagsiekte sheep retrovirus
MMLV	Moloney murine leukemia virus
MMTV	Mouse mammary tumor virus
MPMV	Mason–Pfizer monkey virus
MSRV	Multiple sclerosis-associated retrovirus
OLV	Ovine lentivirus
PDB	Protein Data Bank
PERV	Porcine endogenous retrovirus
PR	Protease
RSV	Rous sarcoma virus
SFV	Simian foamy virus
SIV	Simian Immunodeficiency virus
SMRV	Squirrel monkey retrovirus
SRV-1	Simian retrovirus type 1
SRV-2	Simian retrovirus type 2
STLV-1	Simian T-lymphotropic virus type 1
STLV-2	Simian T-lymphotropic virus type 2
STLV-3	Simian T-lymphotropic virus type 3
VISNA	Maedi visna virus
WDSV	Walleye dermal sarcoma virus
WEHV-1	Walleye epidermal hyperplasia virus type 1
WEHV-2	Walleye epidermal hyperplasia virus type 2
XMRV	Xenotropic murine leukemia virus-related virus
