# Supplementary material for: Dimer Interface Organization is a Main Determinant of Intermonomeric Interactions and Correlates with Evolutionary Relationships of Retroviral and Retroviral-Like Ddi1 and Ddi2 Proteases
Source: Int J Mol Sci. 2020 Feb 17;21(4):1352. doi: 10.3390/ijms21041352 (PMC7072860; doi:10.3390/ijms21041352)
Supplement: Supplementary file 1 [file ijms-21-01352-s001.zip › ijms-706609 supplementaty 1/Table_S1.docx]

**Table S1**. **Dimer stabilities determined experimentally by kinetic assays.** Examples for UC_50_ and K_dapp_ values currently available in the literature are listed. n.o., dissociation was not observed; n.d., not determined. Additional K_d_ values have also been summarized previously for HIV-1 and HIV-2 proteases by Ingr *et al.* [**53**].

| **Protease** | **Enzyme** | **UC50 (M)** | **K_dapp_ (nM)** | **Reference** |
| --- | --- | --- | --- | --- |
| HIV-1 PR | wild-type | 1.47 | 1 | [44] |
|  | wild-type | 1.22 | < 0.37 | [50] |
|  | mutant-G94S | 0.63 | 26.3 ± 11.7 |  |
|  | mutant-T96S | 0.74 | 4.94 ± 1.07 |  |
|  | wild-type | 1.78 | < 10 | [51] |
|  | mutant-L76V | 0.85 | 71 ± 24 |  |
|  | mutant-L24I | 1.05 | 22 | [52] |
|  | mutant-I50V | 0.97 | 19 |  |
|  | mutant-G73S | 1.54 | n.o. |  |
|  | wild-type | n.d. | 15.1 ± 4.3 | [53] |
|  | mutant-T26S | n.d. | 122 ± 28 |  |
| AMV PR | wild-type | n.d. | 412 ± 122 |  |
|  | mutant-S38T | n.d. | 31.5 ± 3.3 |  |
| MPMV PR | wild-type | n.d. | 13.5 ± 8.3 |  |
| HIV-2 PR | wild-type | n.d. | < 1 | [54] |
| XMRV PR | wild-type | 0.20 M | 115 | [44] |
| HFV PR | wild-type | 0.75 M | n.d. | [55] |
| HTVL-1 PR | wild-type, 1-120 residues | n.d. | >3000 | [56] |
|  | wild-type, 1-125 residues | n.d. | 491 |  |

**Reference**

44. Matúz, K.; Mótyán, J.; Li, M.; Wlodawer, A.; Tőzsér, J. Inhibition of XMRV and HIV-1 proteases by pepstatin A and acetyl-pepstatin. *FEBS J.* **2012**, *279*, 3276–3286.

1. Olivares, I.; Mulky, A.; Boross, P.I.; Tőzsér, J.; Kappes, J.C.; Lopez-Galindez, C.; Arias, L.M. HIV-1 Protease Dimer Interface Mutations that Compensate for Viral Reverse Transcriptase Instability in Infectious Virions. *J. Mol. Boil.* **2007**, *372*, 369–381.
2. Louis, J.M.; Zhang, Y.; Sayer, J.M.; Wang, Y.-F.; Harrison, R.; Weber, I.T. The L76V Drug Resistance Mutation Decreases the Dimer Stability and Rate of Autoprocessing of HIV-1 Protease by Reducing Internal Hydrophobic Contacts. *Biochemistry* **2011**, *50*, 4786–4795.
3. Liu, F.; Boross, P.I.; Wang, Y.-F.; Tőzsér, J.; Louis, J.M.; Harrison, R.; Weber, I.T. Kinetic, stability, and structural changes in high-resolution crystal structures of HIV-1 protease with drug-resistant mutations L24I, I50V, and G73S. *J. Mol. Boil.* **2005**, *354*, 789–800.
4. Ingr, M.; Uhlíková, T.; Strísovský, K.; Majerová, E.; Konvalinka, J. Kinetics of the dimerization of retroviral proteases: The “fireman’s grip” and dimerization. *Protein Sci***. 2003**, *12*, 2173–2182.
5. Jordan, S.P.; Zugay, J.; Darke, P.L.; Kuo, L.C. Activity and dimerization of human immunodeficiency virus protease as a function of solvent composition and enzyme concentration. *J. Boil. Chem.* **1992**, *267*, 20028–20032.
6. Fenyofalvi, G.; Bagossi, P.; Copeland, T.D.; Oroszlan, S.; Boross, P.; Tőzsér, J. Expression and characterization of human foamy virus proteinase. *FEBS Lett.* **1999**, *462*, 397–401.
7. Kadas, J.; Boross, P.; Weber, I.T.; Bagossi, P.; Matúz, K.; Tőzsér, J. C-terminal residues of mature human T-lymphotropic virus type 1 protease are critical for dimerization and catalytic activity. *Biochem. J.* **2008**, *416*, 357–364.
