# Supplementary material for: Dimer Interface Organization is a Main Determinant of Intermonomeric Interactions and Correlates with Evolutionary Relationships of Retroviral and Retroviral-Like Ddi1 and Ddi2 Proteases
Source: Int J Mol Sci. 2020 Feb 17;21(4):1352. doi: 10.3390/ijms21041352 (PMC7072860; doi:10.3390/ijms21041352)
Supplement: Supplementary file 1 [file ijms-21-01352-s001.zip › ijms-706609 supplementaty 1/Table_S2.docx]

**Table S2**. **PDB identifiers used for the comparison of contact maps.** Contact maps of the homodimeric enzymes were obtained from PDBsum database. The plotted values are shown in **Figure 3** and **Figure 7**.

| **Protease** | **AMV/RSV** | **MPMV** | **HIV-1** | **HIV-2** | **SIV** | **FIV** | **HTLV-1** | **XMRV** | **Ddi1/Ddi2** |
| --- | --- | --- | --- | --- | --- | --- | --- | --- | --- |
| **PDBID** | 1MVP | 6S1U | 5HVP | 1HII | 1SIV | 2FIV | 3LIY | 4EXH | 2I1A |
|  | 1BAI | 6S1W | 5YOK | 5UPJ | 1TCW | 3FIV | 3LIX | 3SLZ | 4Z2Z |
|  | 2RSP | 6S1V | 7HVP | 6UPJ | 1YTH | 3OGP | 3LIV | 3SM1 | 3S8I |
|  |  |  | 1ZTZ | 2HPE | 1YTG | 3OGQ | 3LIQ | 3SM2 | 4RGH |
|  |  |  | 4LL3 | 3EBZ |  |  | 3LIN | 3NR6 | 5YS4 |
|  |  |  |  |  |  |  | 3LIT |  | 5YQ8 |
|  |  |  |  |  |  |  | 3WSJ |  |  |
|  |  |  |  |  |  |  | 4YDF |  |  |
|  |  |  |  |  |  |  | 4YDG |  |  |
|  |  |  |  |  |  |  | 2B7F |  |  |
