# Supplementary material for: Dimer Interface Organization is a Main Determinant of Intermonomeric Interactions and Correlates with Evolutionary Relationships of Retroviral and Retroviral-Like Ddi1 and Ddi2 Proteases
Source: Int J Mol Sci. 2020 Feb 17;21(4):1352. doi: 10.3390/ijms21041352 (PMC7072860; doi:10.3390/ijms21041352)
Supplement: Supplementary file 1 [file ijms-21-01352-s001.zip › ijms-706609 supplementaty 1/Table_S3.docx]

**Table S3**. **Statistical differences between the contact maps.** PAST v3.26 software was used to plot contact map values (**Figure 3**) and to perform statistical analysis by Mann-Whitney pairwise algorithm (several-sample tests). Raw p values are shown for each studied property, p<0.05 was considered to be statistically significant (highlighted by yellow background).

| **Interface area** | | | | | | | | | |
| --- | --- | --- | --- | --- | --- | --- | --- | --- | --- |
|  | **AMV/RSV** | **MPMV** | **HIV-1** | **HIV-2** | **SIV** | **FIV** | **HTLV-1** | **XMRV** | **Ddi1/Ddi2** |
| **AMV/RSV** |  |  |  |  |  |  |  |  |  |
| **MPMV** | 0.005075 |  |  |  |  |  |  |  |  |
| **HIV-1** | 0.415600 | 0.057490 |  |  |  |  |  |  |  |
| **HIV-2** | 0.302800 | 0.001376 | 0.025690 |  |  |  |  |  |  |
| **SIV** | 0.332900 | 0.002415 | 0.055960 | 0.3986000 |  |  |  |  |  |
| **FIV** | 0.080690 | 0.002362 | 0.8936000 | 0.0233200 | 0.073570 |  |  |  |  |
| **HTLV-1** | 0.000731 | 0.000293 | 0.0002390 | 0.0004700 | 0.001241 | 0.001237 |  |  |  |
| **XMRV** | 0.001376 | 0.001376 | 0.0001820 | 0.0001830 | 0.000449 | 0.000443 | 0.000012000 |  |  |
| **Ddi1/Ddi2** | 0.000462 | 0.000462 | 0.0000278 | 0.0000279 | 0.000101 | 0.000100 | 0.000000382 | 0.01230 |  |

| **No. of interaction residues** | | | | | | | | | |
| --- | --- | --- | --- | --- | --- | --- | --- | --- | --- |
|  | **AMV/RSV** | **MPMV** | **HIV-1** | **HIV-2** | **SIV** | **FIV** | **HTLV-1** | **XMRV** | **Ddi1/Ddi2** |
| **AMV/RSV** |  |  |  |  |  |  |  |  |  |
| **MPMV** | 0.040490 |  |  |  |  |  |  |  |  |
| **HIV-1** | 0.783500 | 0.036740 |  |  |  |  |  |  |  |
| **HIV-2** | 0.826100 | 0.008578 | 0.1181000 |  |  |  |  |  |  |
| **SIV** | 0.360300 | 0.003383 | 0.5881000 | 0.1140000 |  |  |  |  |  |
| **FIV** | 0.647700 | 0.009032 | 0.8920000 | 0.1273000 | 0.7899000 |  |  |  |  |
| **HTLV-1** | 0.214500 | 0.000942 | 0.0789300 | 0.0012520 | 0.0337400 | 0.0552600 |  |  |  |
| **XMRV** | 0.162300 | 0.698000 | 0.0104800 | 0.0089820 | 0.0012910 | 0.0052360 | 0.000120000 |  |  |
| **Ddi1/Ddi2** | 0.000376 | 0.000379 | 0.0000232 | 0.0000235 | 0.0000835 | 0.0000832 | 0.000000304 | 0.01321 |  |

| **Hydrogen bonds** | | | | | | | | | |
| --- | --- | --- | --- | --- | --- | --- | --- | --- | --- |
|  | **AMV/RSV** | **MPMV** | **HIV-1** | **HIV-2** | **SIV** | **FIV** | **HTLV-1** | **XMRV** | **Ddi1/Ddi2** |
| **AMV/RSV** |  |  |  |  |  |  |  |  |  |
| **MPMV** | 0.07220 |  |  |  |  |  |  |  |  |
| **HIV-1** | 0.03466 | 0.17190 |  |  |  |  |  |  |  |
| **HIV-2** | 0.03466 | 0.03466 | 0.59470 |  |  |  |  |  |  |
| **SIV** | 0.04768 | 0.84540 | 0.13660 | 0.03343 |  |  |  |  |  |
| **FIV** | 0.04768 | 0.04768 | 0.89910 | 0.52240 | 0.053010 |  |  |  |  |
| **HTLV-1** | 0.01333 | 0.04026 | 0.95080 | 0.80510 | 0.018690 | 0.35260 |  |  |  |
| **XMRV** | 0.03466 | 0.03466 | 0.01167 | 0.01167 | 0.018940 | 0.01894 | 0.002531 |  |  |
| **Ddi1/Ddi2** | 0.01810 | 0.01810 | 0.00632 | 0.01010 | 0.008134 | 0.01030 | 0.000593 | 0.1217 |  |

| **Non-bonded contacts** | | | | | | | | | |
| --- | --- | --- | --- | --- | --- | --- | --- | --- | --- |
|  | **AMV/RSV** | **MPMV** | **HIV-1** | **HIV-2** | **SIV** | **FIV** | **HTLV-1** | **XMRV** | **Ddi1/Ddi2** |
| **AMV/RSV** |  |  |  |  |  |  |  |  |  |
| **MPMV** | 0.08086 |  |  |  |  |  |  |  |  |
| **HIV-1** | 0.55100 | 0.03689 |  |  |  |  |  |  |  |
| **HIV-2** | 0.29380 | 0.37110 | 0.021570 |  |  |  |  |  |  |
| **SIV** | 0.59590 | 0.05183 | 0.270300 | 0.11130 |  |  |  |  |  |
| **FIV** | 0.85970 | 0.05183 | 0.019960 | 0.39130 | 0.03038 |  |  |  |  |
| **HTLV-1** | 0.93260 | 0.01425 | 0.008458 | 0.12580 | 0.22930 | 0.22930 |  |  |  |
| **XMRV** | 0.03689 | 0.03689 | 0.012190 | 0.01219 | 0.01996 | 0.01996 | 0.002694 |  |  |
| **Ddi1/Ddi2** | 0.01863 | 0.01863 | 0.004257 | 0.004257 | 0.00836 | 0.00836 | 0.000446 | 0.1238 |  |
