# Supplementary material for: Dimer Interface Organization is a Main Determinant of Intermonomeric Interactions and Correlates with Evolutionary Relationships of Retroviral and Retroviral-Like Ddi1 and Ddi2 Proteases
Source: Int J Mol Sci. 2020 Feb 17;21(4):1352. doi: 10.3390/ijms21041352 (PMC7072860; doi:10.3390/ijms21041352)
Supplement: Supplementary file 1 [file ijms-21-01352-s001.zip › ijms-706609 supplementaty 1/Table_S4.docx]

**Table S4**. **Uniprot identifiers of the studied proteases.**

| **Protease group** | **Virus / Organism** | **UniProt ID** |
| --- | --- | --- |
| **Lentivirus** | HIV-1 | P12497 |
|  | HIV-2 | P04584 |
|  | SIV | P05896 |
|  | EIAV | P32542 |
|  | FIV | Q66972 |
|  | OLV | P16901 |
|  | VISNA | P23427 |
|  | CAEV | P33459 |
|  | BIV | P19560 |
| **Spumaretrovirus** | EFV | Q9J4C7 |
|  | BFV | Q8ALU1 |
|  | FFV | O93209 |
|  | HFV | P14350 |
|  | SFV | P23074 |
| **Alpharetrovirus** | AMV | P26315 |
|  | RSV | P03322 |
| **Deltaretrovirus** | HTLV-1 | Q82134 |
|  | HTLV-2 | P03353 |
|  | HTLV-3 | Q0R5R3 |
|  | BLV | P10270 |
|  | STLV-1 | Q4QY52 |
|  | STLV-2 | O70641 |
|  | STLV3 | Q8URT2 |
| **Epsilonretrovirus** | WEHV-1 | Q9WHI7 |
|  | WEHV-2 | Q9WHJ2 |
|  | WDSV | O92815 |
| **Gammaretrovirus** | FELVP | 10273 |
|  | GALV | P21414 |
|  | MMLV | P03355 |
|  | XMRV | Q9E7M1 |
|  | BAEV | P10272 |
|  | PERV | Q9Q1X5 |
| **Betaretrovirus** | MPMV | P07570 |
|  | MMTV | P10271 |
|  | SRV-1 | P04024 |
|  | SRV-2 | P51518 |
|  | JSRV | P31625 |
|  | SMRV | P21407 |
| **Non-viral**  **(Ddi1/Ddi2)** | Ddi1-Sc | P40087 |
|  | Ddi1-Hs | Q8WTU0 |
|  | Ddi2-Hs | Q5TDH0 |
|  | Ddi1-Lm | I7HUG0 |
