# Supplementary figures and images for: Dimer Interface Organization is a Main Determinant of Intermonomeric Interactions and Correlates with Evolutionary Relationships of Retroviral and Retroviral-Like Ddi1 and Ddi2 Proteases
Source: Int J Mol Sci. 2020 Feb 17;21(4):1352. doi: 10.3390/ijms21041352 (PMC7072860; doi:10.3390/ijms21041352)

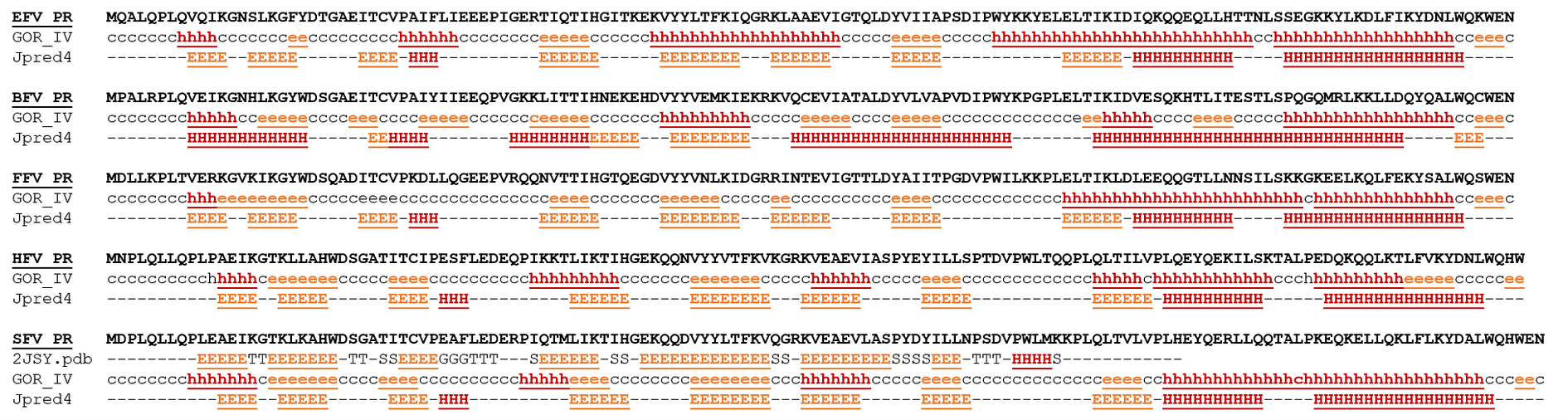

Supplement: Supplementary file 1 [file ijms-21-01352-s001.zip › ijms-706609 supplementaty 1/Figure_S1.png]

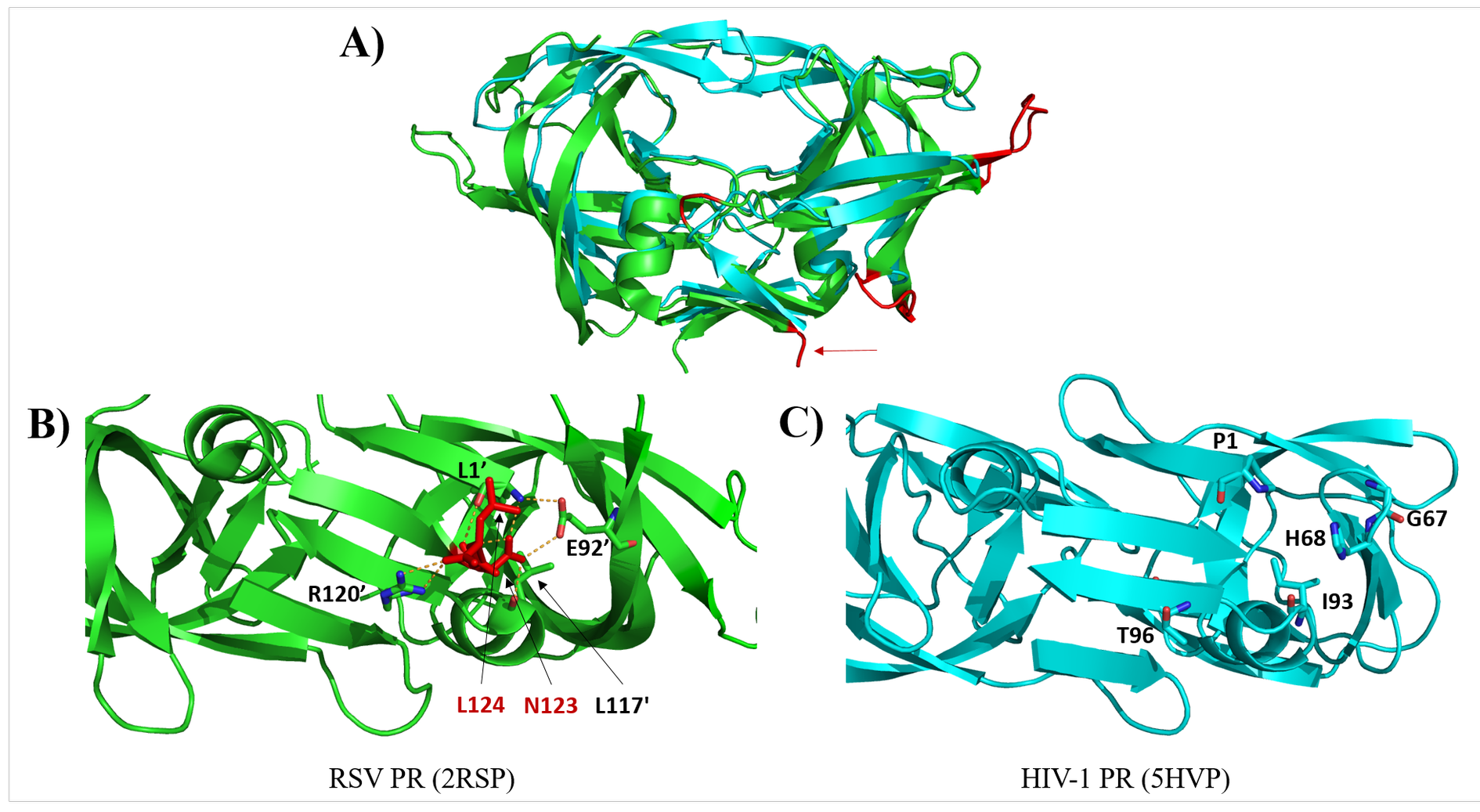

Supplement: Supplementary file 1 [file ijms-21-01352-s001.zip › ijms-706609 supplementaty 1/Figure_S2.png]

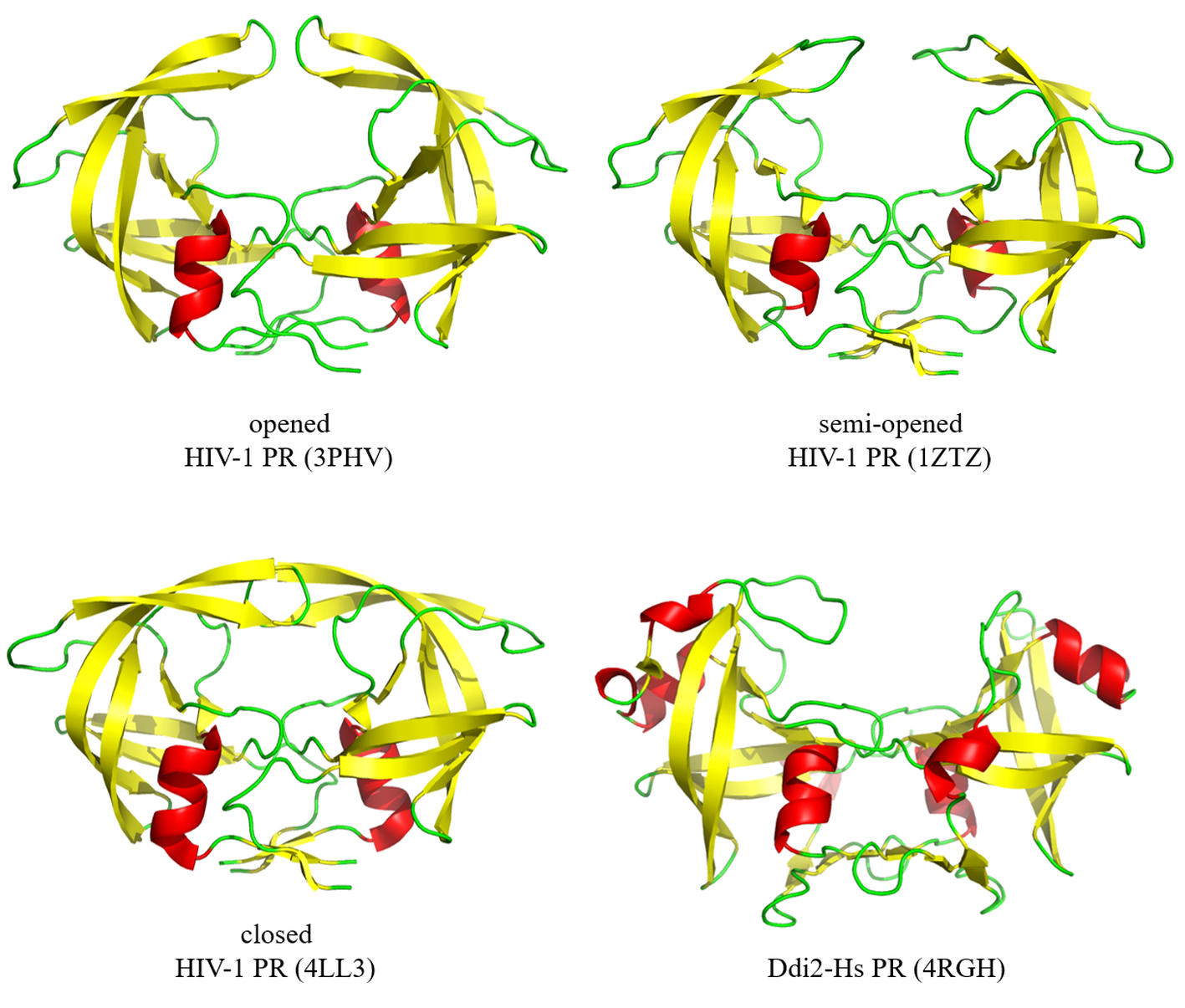

Supplement: Supplementary file 1 [file ijms-21-01352-s001.zip › ijms-706609 supplementaty 1/Figure_S3.png]
